# Pennsylvania

**Published:** 1897-03

**Authors:** 


*Pennsylvania.* Thirty head of tubercular cattle were recently killed at the Schuylkill County Almshouse. Nineteen thoroughbred Holstein-Friesians were destroyed in Berks County.

